# Organic and Inorganic Modifications to Increase the Efficiency in Immobilization of Heavy Metal (Zn) in Cementitious Composites—The Impact of Cement Matrix Pore Network Characteristics

**DOI:** 10.3390/ma17215281

**Published:** 2024-10-30

**Authors:** Maciej Kalinowski, Karol Chilmon, Jan Bogacki, Piotr Woyciechowski

**Affiliations:** 1 Faculty of Civil Engineering, Warsaw University of Technology, Al. Armii Ludowej 16, 00-637 Warsaw, Poland; maciej.kalinowski@pw.edu.pl (M.K.); karol.chilmon@pw.edu.pl (K.C.); piotr.woyciechowski@pw.edu.pl (P.W.); 2 Faculty of Building Services, Hydro and Environmental Engineering, Warsaw University of Technology, Nowowiejska 20, 00-653 Warsaw, Poland

**Keywords:** cementitious composites, heavy metal immobilization, organic shells, iron slag, water treatment

## Abstract

This research investigated the properties of modified cementitious composites including water purification from heavy metal—zinc. A new method for characterizing the immobilization properties of tested modifiers was established. Several additions had their properties investigated: biochar (BC), active carbon (AC), nanoparticulate silica (NS), copper slag (CS), iron slag (EAFIS), crushed hazelnut shells (CHS), and lightweight sintered fly ash aggregate (LSFAA). The impact of modifiers on the mechanical and rheological properties of cementitious composites was also studied. It was found that considered additions had a significantly different influence over the investigated properties. The addition of crushed hazelnut shells, although determined as an effective immobilization modifier, significantly deteriorated the mechanical performance of the composite as well as its rheological properties. Modification by iron slag allowed for a significant increase in immobilization properties (five-fold compared to the reference series) without a substantial impact on other properties. The negative effect on immobilization efficiency was observed for nanoparticulate silica modification due to its sealing effect on the pore network of the cement matrix. The capillary pore content in the cement matrix was identified as a parameter significantly influencing the immobilization potential of most considered modifications, except biochar and active carbon.

## 1. Introduction

Depending on the type of modification, cementitious composites can be characterized by various properties other than purely mechanical. Through the addition of internal curing agents, concrete gains the ability to self-repair [1,2,3] (prolonging durability and reducing the need for an element’s repair or its replacement due to mechanical failure). By adding photocatalytic materials, both in the entire volume or as a surface modification, concrete can purify surrounding air from gaseous pollutants (both organic and inorganic) driven by specific electromagnetic radiation, including solar radiation [4,5,6,7]. There are other modifiers that allow concrete to be electrically conductive or chem-resistant [8,9,10]. The common factor between all those cementitious composites is that they represent passive solutions, requiring no human-made energy to operate, and can be implemented on an industrial scale to improve certain negative environmental consequences of urban development. Although it is still not viable to say that those techniques can completely replace other developed active systems (water purification stations, waste processing units, and others), they can act as supplementary systems, contributing to an improvement of various environmental factors over large areas.

Most surfaces (sidewalks, roads) are exposed to rainfall, which collects different pollutants of various origins, both organic and inorganic. By including in the composition of concrete additions characterized with immobilization properties, the quality of the surface runoff can then improve, reducing the risk of groundwater contamination [11]. In this context, the authors explored the viability of using modified cementitious composites as part of a sequential water treatment system designed to reduce heavy metal concentrations in surface runoff.

As rainfall water flows over the concrete’s external surface (regular concrete) or within its entire volume (pervious concrete), physical and chemical phenomena contribute to immobilizing pollutants of various origins within the cement matrix [12,13]. Most studies have been conducted for pervious concretes, as they represent cementitious composites that allow for a dynamic flow of liquids through its vast and interconnected pore network [14,15,16,17].

Pollution should be eliminated from the environment whenever possible. The most troublesome and chemically persistent water pollutants include heavy metals like zinc, copper, lead, cadmium, chromium, mercury, and others [18]. For the purposes of this study, due to its high environmental concentrations and impact on living organisms, zinc was selected as a model pollutant. It is present in various products and industrial processes such as steel production, metal galvanization, battery manufacturing, paints, fertilizers, and pharmaceuticals [19]. Emissions from these processes can introduce significant amounts of zinc into the aquatic environment, both in dissolved and solid forms. Estimated zinc concentrations in surface runoff range from several micrograms to tens per liter [20]. However, in the first portions of rainwater rinsing impermeable surfaces, zinc concentrations may be much higher, up to several milligrams per liter [21]. Although zinc is an essential trace element for living organisms and may occur naturally in the environment in specific amounts, an excess of this element can adversely affect aquatic ecosystems and human health [22]. Zinc pollution can disrupt aquatic organisms’ metabolic processes, alter aquatic ecosystems’ structures or functioning, and degrade drinking water quality [23,24].

**Figure 1:**
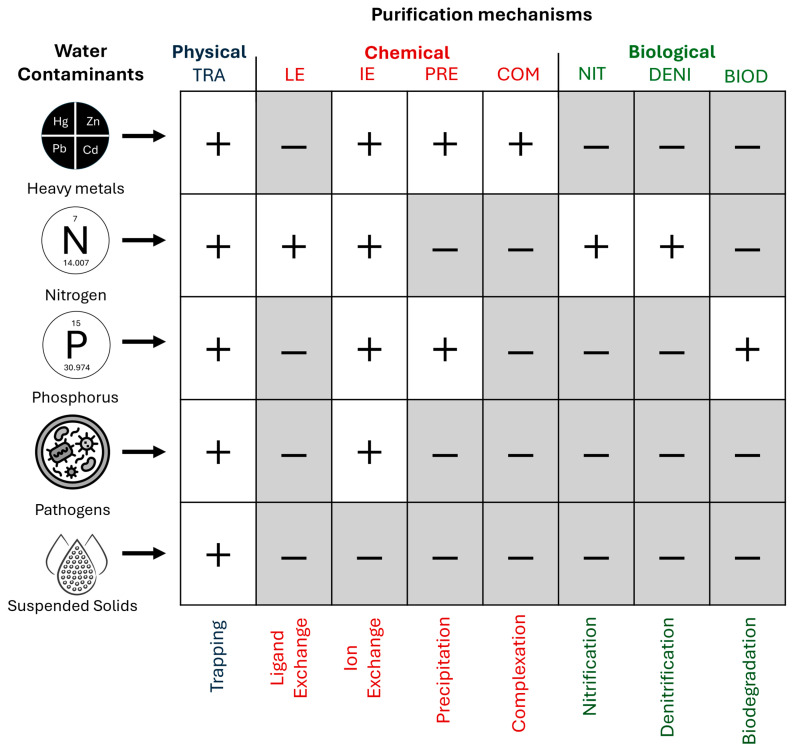
Purification mechanisms from contaminants of various origins (acronyms describing the types of mechanisms: TRA—trapping, LE—ligand exchange, IE—ion exchange, PRE—precipitation, COM—complexation, NIT—nitrification, DENI—denitrification, and BIOD—biodegradation).

Water purification mechanisms can vary depending on the material and environmental conditions (Figure 1). The mechanisms themselves are passive and do not require chemicals or energy [25]. Heavy metals could be retained at the liquid–solid interface using adsorption mechanisms [26]. Some organic compounds can form complexes with heavy metals, creating stable chemical compounds that are less toxic and easier to remove from aqueous solutions [8,27]. Different functional groups on grains’ surfaces can interact with zinc ions in many ways, e.g., electrostatic interaction, ion exchange, and diffusion [28].

The cement matrix could be considered to act as a ceramic membrane for metal removal. The contaminated solution enters the internal solid’s structure, which causes ionic capture within the material pore network, which can reduce their mobility and availability to living organisms. However, the membrane removal of substances at the level of simple ions requires minimum pore sizes typical of nanofiltration or reverse osmosis [29]. Moreover, organic compounds may contain functional groups capable of chemically reducing or oxidizing heavy metal ions, leading to changes in their solubility [25]. Finally, purely physical filtration and separation may occur. This mechanism can be applied in concrete, where heavy metals can be mechanically retained within the material structure, allowing for their separation [30]. In practice, it could be expected that the overall removal mechanism will be a combination of different phenomena.

During pollutant contact with cementitious composite, due to the alkaline environment of the cement matrix, heavy metal ions precipitate (the solubility of heavy metal ions is negatively correlated with an increase in pH) [31] and then are adsorbed on the surface of the cement matrix.

As with any other, this property of cementitious composites can be enhanced. As cement matrix properties are the driving force behind concrete immobilization potential, applying changes to its characteristics (mainly regarding pore network, density, and chemical composition), different mechanisms through which filtration occurs can be significantly altered [32]. Differences in binder composition can impact the alkalinity of the composite, influencing its overall pH and directly affecting immobilization efficiency [8]. As the water-to-cement ratio changes, so does the porosity of the matrix, contributing to yet another varying factor in immobilization efficiency—the trapping potential.

The composition of the concrete mix can also include various modifiers characterized by immobilization properties used in other filtration applications [33]. Most have a particular grain morphology and pore network characteristics—high specific surface area and an extensive network of interconnected pores of small diameters. Organic modifiers include those usually used for filtration: active carbon, biochar, organic polymers, or different plant-based granulated materials (shells, husks, and others) [34]. Due to their unique structure, the surface that can be used for adsorption is exceptionally high, allowing for high-efficiency immobilization even in the case of a dynamic flow of polluted water through such a system.

Inorganic materials can also be used for that purpose—the addition of metal oxides in different forms (nanoparticles, iron-oxide coated aggregate, iron slag, copper slag), zeolites, nano-silica, and others can provide significant changes to the specific surface of the cement matrix, altering its immobilization properties as well as fluid diffusion characteristics [35,36]. It is worth noting that materials that are by-products of numerous industrial processes with limited application potential demonstrate immobilization properties. Those include organic and inorganic materials produced in vastly different parts of the economy (metallurgy, food processing, and energy generation). Its use in concrete technology would allow new large-scale utilization schemes and provide a solution to reach a circular economy.

Immobilization effectiveness depends heavily on the compatibility between the modifier and cement matrix or, rather, the lack thereof. For adsorption and other phenomena associated with it to occur, the modifier’s grains must be directly exposed to pollutants. If this is not the case and modifier grains are fully integrated with the cement matrix, entirely covered with hydration products, the properties and characteristics of the cement matrix have a profuse influence over composite performance regarding the aforementioned immobilization process. This issue can be overcome to some extent in the case of non-pervious cementitious composites, where different methods of alterations to surface grain exposure and roughness can be applied during the production process. However, regarding rainwater management, pervious concrete represents a more efficient and eco-friendly solution, allowing for improvements in various aspects of it, especially regarding water retention efforts. The exposure of the modifier within the entire volume of such composite is much more challenging to provide, alongside scaling those methods for industrial applications.

### Research Significance

The authors decided to investigate the purposefulness of using various materials as partial replacements of fine aggregate to increase the efficiency of cementitious composites in zinc immobilization. Although some studies have been performed on the issue [13,16,32,34,36,37], including more than a few modifier types in single research is uncommon.

The research focused on both organic and inorganic modifiers, as well as mortars modified with these materials. The organic modifiers used were activated carbon, biochar, and crushed hazelnut shells. Activated carbon and biochar are commonly used in industrial filtration processes due to their highly developed porous structure and rough surface texture, which facilitate the physical entrapment of heavy metal ions. Crushed hazelnut shells were selected for their structural similarities to activated carbon and biochar and a production process that does not require thermal decomposition, with the goal of exploring potential applications for this waste product in construction materials.

The inorganic modifiers included electric arc furnace slag, nanoparticulate silica, lightweight fly ash aggregate, and copper slag. Nanoparticulate silica was selected due to its well-documented effectiveness in modifying the pore structure of cementitious composites. It was used primarily to assess how reducing porosity could influence the composite’s ability to adsorb zinc ions. Electric arc furnace slag and copper slag were incorporated because of their iron oxide content, which can react with zinc ions to form stable compounds, thereby reducing zinc mobility within the cement matrix. Lightweight fly ash aggregate was included primarily due to its porous structure, which offers additional physical sites for zinc immobilization, and its potential pozzolanic reactivity, which could generate additional calcium silicate hydrate (C-S-H) phases around the aggregate particles, thereby increasing the chemical binding sites available for zinc adsorption.

It has been reported that specific modifiers can severely impact the performance of concrete, contributing to a deterioration in mechanical properties and resistance to corrosive agents [38,39,40,41,42]. Due to this, authors decided on a comprehensive approach to the issue, focusing equally on the effect of applied modifications on both heavy metal immobilization potential as well as on other properties of cementitious composites, allowing for the identification of negative impacts of various modifiers and, consequently, development of methods and protocols allowing for their mitigation.

## 2. Materials and Methods

### 2.1. Materials

In the performed research, several different additions were considered: electric arc furnace air-cooled iron slag (EAFIS, ArcelorMittal Warsaw, Warsaw, Poland)—a by-product of melting and refining iron scrap metals; copper slag (CS, KOS Korund, Koło, Poland)—non-ferrous metal slag, a by-product of copper extraction by smelting; activated carbon (AC, type—BA 10, Elbar Katowice, Katowice, Poland)—adsorbent used in various filtration applications produced from hard coal using the steam gas method; biochar (BC, Treeden Group, Lublin, Poland)—a carbon-rich material, a by-product of pyrolysis process during the thermochemical decomposition of biomass from sunflower husk, wood pellets, and wood chips; nanoparticulate silica (NS, Łaziska Steelworks, Łaziska, Poland)—a by-product obtained during the production of metallic silicon and ferrosilicon alloys in arc furnaces; lightweight sintered fly ash aggregate (LSFAA, LSA LLC, Białystok, Poland)—a product of high-temperature sintering (1000–1200 °C) of fly ash produced from the combustion of hard coal in coal boilers; and crushed hazelnut shells (CHS, locally sourced material from a marketplace, Warsaw, Poland)—an organic by-product from nut processing plants.

Two modifiers (CHS and EAFIS) were ground mechanically to different granulations, as they were acquired as coarse granulated materials. In the case of CHS, two variants were considered in the research: 0/1 and 1/2 mono-fractions. EAFIS was initially ground to 2/4, 1/2, and 0/1 fractions.

**Figure 2:**
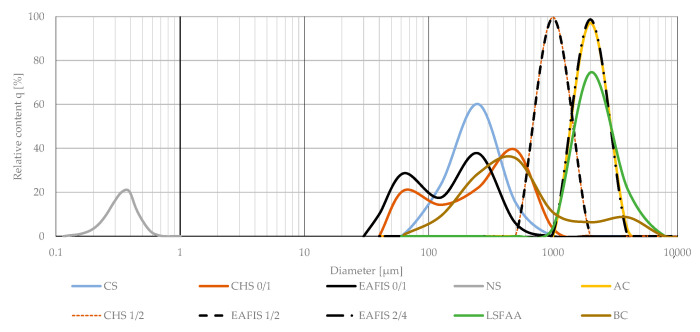
Granulation of modifiers used in the study, both fine and coarse-graded. Modifiers of the same origin, but varying in the degree of refinement are presented with the same color, differing in the line type (continuous and dashed lines).

The granulation of all fine-graded modifiers varied significantly. A bimodal grain size distribution of fine-graded CHS and EAFIS could be observed for materials refined in the laboratory, a typical characteristic of any mechanical refinement process (Figure 2).

**Figure 3:**
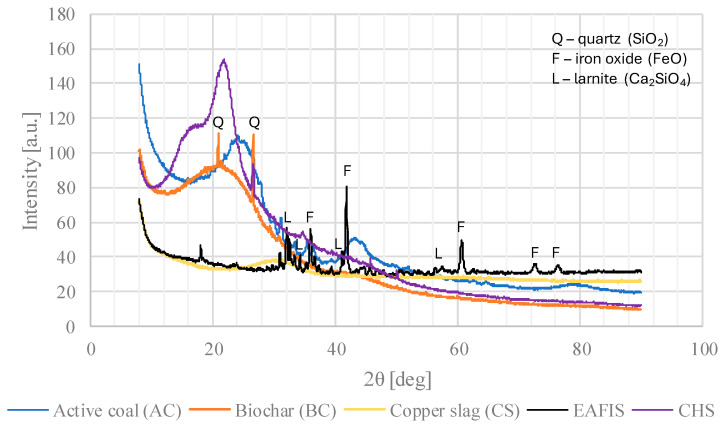
XRD analysis for considered modifications, with selected peaks indicating crystalline structures (SiO_2_, FeO, and Ca_2_SiO_4_)—peaks indicate a crystalline structure of the material (EAFIS), while their lack indicates an amorphous structure (CS, CHS, BC, and AC).

The qualitative phase composition of AC, BC, CS, EAFIS, and NS was investigated using the XRD method (Figure 3). Strong peaks indicating the presence of multiple carbon phases could be noticed around 25° and 43° in the case of AC and 22° in the case of BC. Peaks with different diffraction intensities from quartz could be observed in all samples except biochar (BC). The presence of quartz in the case of AC was probably due to contamination introduced during the storage, transport, or technological processes to which these materials were subjected (grinding and others). No peaks from crystalline phases could be observed in copper slag, whereas EAFIS consisted primarily of crystalline phases, such as crystalline iron oxide, larnite, mayenite, and calcium magnesium aluminum silicate. The difference in phase composition between the two slags (EAFIS and CS) was probably caused by different cooling methods. Air-cooled slags, such as EAFIS, are characterized by the crystalline structure, while water-cooled slags consist mainly of the amorphous phase. This led to the conclusion that CS slag was obtained using the wet method, which is standard in the copper processing industry.

**Figure 4:**
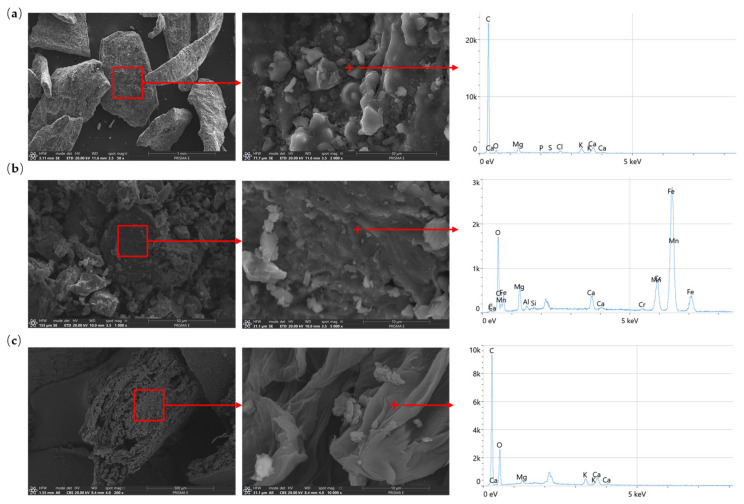
Morphology and chemical composition (SEM/EDS) of (**a**) BC (biochar), (**b**) EAFIS 0/1 (electric arc furnace iron slag), and (**c**) CHS 1/2 (crushed hazelnuts shells); micrographs obtained with the use of PRISMA E Scanning Electron Microscope (Thermo Fisher Scientific, Waltham, MA, USA) with an EDS X-ray microanalyzer.

The morphology of modifier grains varied depending on the material’s origin and preparation technology (Figure 4). EAFIS, BC, and CHS were characterized by non-regular grain morphology. Additionally, all considered organic materials (BC and CHS) were characterized by extensive pore networks of varying diameters and organization within the grain’s volume. Electric arc furnace iron slag (EAFIS) had a significantly denser structure than BC and CHS. Biochar (BC) and crushed hazelnut shells (CHS) consisted mainly of carbon; small quantities of magnesium, sodium, potassium, and calcium could be noticed in the EDS spectra of BC, which could be explained by the unburnt parts of biomass. The strong presence of iron could be noticed in the EAFIS specimen among chromium, manganese, calcium, magnesium, and aluminum.

Ordinary Portland Cement CEM I 42.5 R (compliant with PN-EN 197-1 [43] standard, Heidelberg Materials Poland, Górażdże, Poland) was used to prepare cementitious composites. CEM I-type cement was chosen to reduce the number of independent variables in the experiment (the influence on non-clinker binder ingredients in other types of cement). Its properties were investigated regarding mechanical performance (PN-EN 196-1 [44]), initial and final setting times (PN-EN 196-3 [45]), as well as the specific gravity and specific surface area measured (PN-EN 196-6 [46])—Table 1. Mechanical performance was investigated for cement mortars prepared according to the aforementioned standard, with a water-to-cement ratio of 0.50 and a cement-to-fine aggregate ratio of 3.0. Initial and final setting times were investigated for cement paste of standard consistency (water-to-cement ratio of 0.256) measured with an automatic Vicat apparatus (Controls Vicamatic, Milano, Italy). Specific gravity was investigated for cement using Le Chatelier’s flask according to the aforementioned standard. Its specific surface area was measured using the Blain apparatus and correlated to the specific surface area of a reference cement.

Vistula river sand 0/2 was used as a fine aggregate. Its properties were confirmed according to various standards—PN-EN 933-1 (granulation) [47], PN-B-06714-46 (alkali-silica reaction) [48], and PN-EN 1744-1 (organic pollutants content) [49], among others. The water used in this study met the requirements of EN 1008 [50]. An additional mass of superplasticizer (aqueous polycarboxylate solution) was added to cementitious mortars to modify their rheological properties. It met the PN-EN 934-2 standard requirements [51] and was characterized by electrostatic and steric mechanisms of action.

### 2.2. Experimental Framework

**Figure 5:**
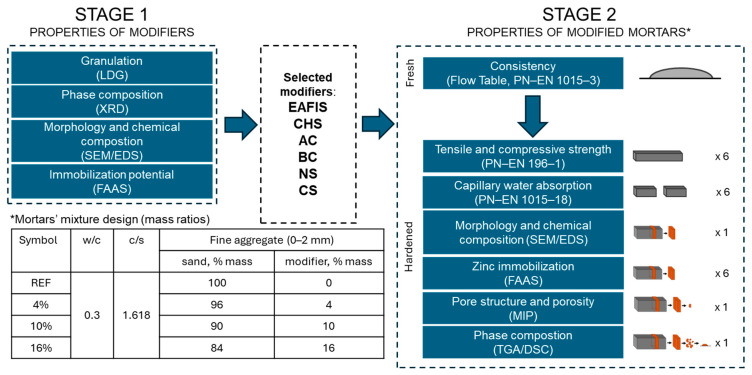
The experimental framework.

The designed experiment was divided into two main stages (Figure 5). In the first phase, the properties of all considered modifications were investigated, including zinc immobilization efficiency. As most of the considered modifiers were not characterized by binding properties, the modification of cement mortars involved the replacement of a portion of filler material (fine aggregate) with an increasing mass amount of immobilization modifiers. The binder content in all prepared sample series remained the same, along with the water-to-cement ratio equal to 0.30 and the cement-to-sand ratio of 1.618. This approach was established to maintain the same quality and quantity of non-modified cement matrix as in a reference series in all other modified mortars.

In the modified series, a part of the fine aggregate mass was replaced with the modifier’s mass: 4%, 10%, and 16%, respectively. Prismatic mortar samples with dimensions of 40 mm × 40 mm × 160 mm, varying in the mass content of the modifier, were prepared for each of the investigated modifiers in stage 2. For each series, fifteen samples were prepared. Six samples from each series were used to test the mechanical properties of the mortars, another six for the capillary water adsorption test, and two for the zinc immobilization test. One sample was used for phase composition analysis and mercury intrusion porosimetry. SEM/EDS analysis was performed to investigate zinc immobilization on the surface of the investigated composites, as well as the compatibility of the modifier’s grains with the cement matrix.

### 2.3. Methods

**Figure 6:**
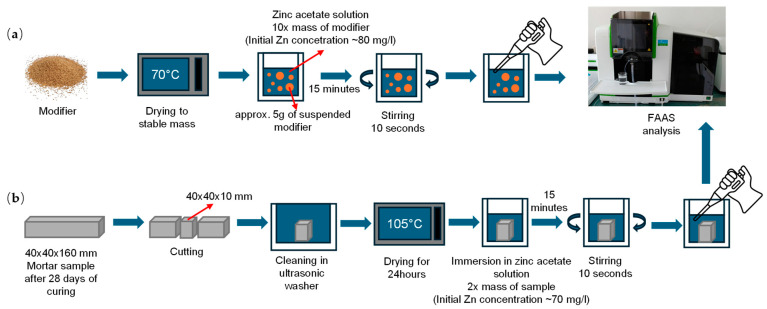
The experimental procedure to investigate the immobilization potential of investigated (**a**) modifiers and (**b**) mortars.

The immobilization potential of investigated modifiers was established according to the protocol developed by the authors (Figure 6). After drying at 70 °C until its mass stabilized, approx. 5 g of each studied material was placed in a 100 mL beaker and immersed for 15 min in zinc acetate solution. The mass of the solution varied between samples and equaled ten times the mass of an individual sample. At least three samples were prepared for each of the considered modifications. After 15 min of sample immersion, the solution was stirred, and a portion of it, after filtration through a paper filter (graded to a degree preventing any solution contamination with modifiers grains), was transferred to a 50 mL bottle. The authors decided on a fixed time of sample exposure to the zinc environment to establish similar conditions for all investigated samples. Although exposure time could be shorter (the phenomena linked with surface adsorption mostly occur directly after exposure to contaminated medium), to avoid unnecessary differences that could impact the quality of the experiment, the authors decided to prolong the test procedure to 15 min. That way, any variation in exposure time would not significantly impact the quality of the experiment.

The collected solution was used to determine the change in zinc concentration due to exposure to cementitious composite via flame atomic absorption spectrometry (FAAS, PinAAcle 900F PerkinElmer, Waltham, MA, USA). A reference sample of zinc acetate solution (not exposed to the modifier) was tested for each series, allowing control over the change in reference zinc concentrations between tests.

Cementitious composites, after preparation and rheological tests performed according to PN-EN 1015-3 standard [52], were molded and stored for 24 h in a curing chamber (temperature 20 ± 2 °C, relative humidity RH ≥ 95%). Afterward, samples were demolded and cured in the curing chamber until further testing.

Mechanical properties (tensile and compressive strength) were investigated according to the PN-EN 196-1 [44]. Three prism-shaped specimens with dimensions of 40 mm × 40 mm × 160 mm (with the latter halves of those prisms remaining after the three-point bending test) were prepared for tests after 7 and 28 days from sample preparation.

Capillary water absorption tests were performed according to PN-EN 1015-18 [53]. After 28 days of curing, the aforementioned prism-shaped specimens were covered with a layer of epoxy resin. After it hardened, the samples were broken into two parts of roughly the same volume and dried at 40 °C until their mass stabilized. Next, the prepared samples were placed in a tray with distilled water that allowed the fracture surface of each half of the specimen to be immersed in water 5–10 mm above the highest point on the base of the specimen. The experiment started with the samples’ contact with water and measured its time-dependent mass change after 10 min, 90 min, and 24 h. Before each weighing, the excess water adhered to the test specimen was removed with a dry cloth. The water absorption by partial immersion indicated the mass of water absorbed per area due to capillary suction forces. Based on the test results, two values characterizing the absorption potential of cementitious composites were calculated according to the formulas included in the aforementioned standard.

The efficiency of zinc immobilization by cementitious composites was examined according to the protocol developed by the authors. After 28 days of curing, prism-shaped mortar samples were mechanically cut into smaller prisms with dimensions of 40 mm × 40 mm × 10 mm. Then, the cut samples were placed in an ultrasonic washer for 15 min to remove any dust from its surface that originated from sample preparation. Afterward, the samples were dried at 105 °C for 24 h. Next, the samples were placed in ambient lab conditions to cool down, weighted and put in 100 mL beakers on the side with a smaller surface area. Zinc acetate solution was then added to the beakers. Its mass varied between samples and equaled double the sample mass. After 15 min of sample immersion, the solution was stirred, and a portion of it was transferred to a 50 mL bottle.

The collected solution was used to determine the change in zinc concentration due to exposure to cementitious composite via flame atomic absorption spectrometry (FAAS, PinAAcle 900F PerkinElmer, Waltham, MA, USA). For each series, six samples were prepared. A reference sample of zinc solution (not exposed to cementitious composites) was tested for each series, allowing control over the change in reference zinc concentrations between tests.

Mercury intrusion porosimetry tests were performed to investigate the influence of different modifiers over the pore network characteristics of cementitious composites with Poremaster 33 (Quantachrome Instruments, Inc., Boynton Beac, FL, USA). Prismatic samples of the same size and preparation protocol as in the heavy metal immobilization test case were prepared. Before the mercury intrusion, the samples were crushed to obtain a small enough mortar specimen to fit into the penetrometer. The mass of any individual sample was recorded. The mercury intrusion was performed on the low-pressure station (pressure up to 55 psi) and high-pressure station (pressure up to 33,000 psi), allowing for the receipt of various characteristics of the pore network in the investigated cementitious composites in the pore diameter range of 1100 to 0.0064 µm. Knowing the volume of intruded mercury, the samples’ specific and apparent densities were also calculated.

Thermal analysis methods were utilized to analyze the thermal degradation process of the prepared cementitious composites. Prismatic samples of the same size and preparation protocol as in the heavy metal immobilization test case were prepared. Afterward, samples were crushed and ground to obtain a fine-graded sample for thermal analysis tests. An SDT Q600 V20.9 Build 20 Thermal Gravimetric Analyzer (TA Instruments, New Castle, DE, USA) was used to perform tests. The test commenced after powder samples were placed in an alumina pan in a synthetic air environment. The change in the samples’ mass and heat flow as the temperature increased from 25 to 1000 °C with an increase of 5 °C/min was measured.

All SEM micrographs were obtained using a PRISMA E Scanning Electron Microscope (Thermo Fisher Scientific, Waltham, MA, USA) with an EDS X-ray microanalyzer. The samples for microscopic imagery were spurred with either gold or carbon.

## 3. Results and Discussion

### 3.1. Zinc Immobilization Efficiency of Considered Modifiers

The performed study began with an initial determination of the immobilization properties of several different modifiers. The efficiency in immobilizing zinc was investigated, as it is one of the most common heavy metal surface runoff contaminants [54,55]. In all performed tests, its concentration in the solution before its placement in the presence of the modifier was at least 70 mg/L—well above the expected Zn concentration in surface runoff (which is highly dependent on the sample collection location and can reach several milligrams per liter in urban surface runoff [21]). However, it was decided to investigate the immobilization potential at high heavy metal concentrations to establish the immobilization efficiency in extreme conditions. Furthermore, higher concentrations of zinc ions enabled a more detailed analysis of the specimens using TGA and SEM techniques. The increased concentrations enhanced the likelihood of detecting the incorporation of zinc compounds in the structure of the examined materials and helped minimize the risk of measurement errors that could arise with lower zinc concentrations.

**Figure 7:**
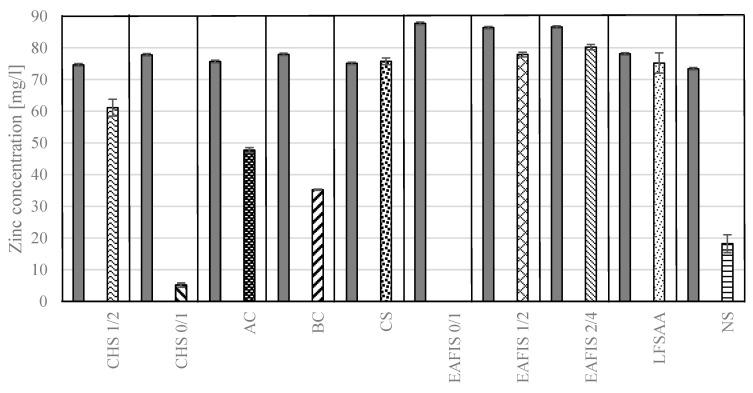
The concentration of zinc in solutions exposed to modifier grains for 15 min and its reference value in the solution before the samples’ immersion for each of the performed tests.

It was found that most of the considered materials exhibit the ability to significantly reduce zinc concentration in the solution (Figure 7). The most effective modifier was electric arc furnace iron slag of granulation 0/1. In this case, the zinc concentration in the solution dropped from approx. 85 mg/L to 0 mg/L after 15 min of exposure to the modifier’s grains. A comparable efficiency was observed for crushed hazelnut shells of 0/1 granulation—a reduction from 78 mg/L to approx. 5 mg/L was observed with the same exposure time. Nanoparticulate silica also displayed significant immobilization properties, reducing the zinc content from 75 mg/L to approx. 18 mg/L. Other modifiers’ abilities to immobilize had a lesser effect. The least effective were copper slag, LFSAA, and iron slag of coarser granulations. In the cases of copper slag and LFSAA, no statistically significant (ANOVA, *p* > 0.05) reduction was observed.

**Figure 8:**
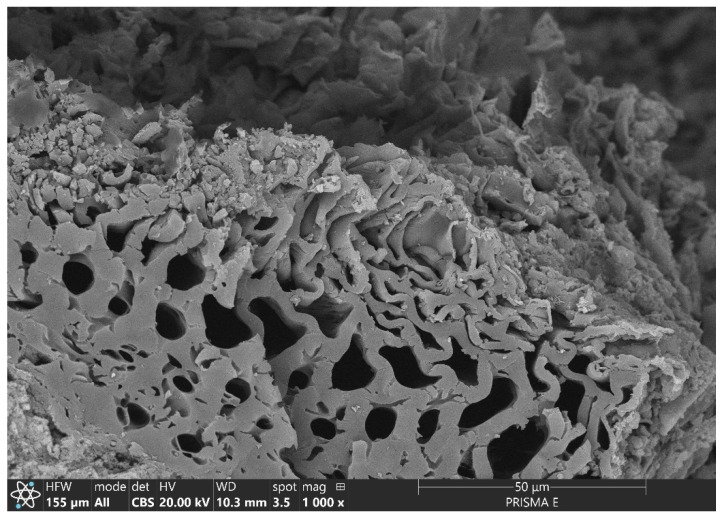
The morphology of biochar grain with its internal extensive pore network exposed to the heavy metal solution, increasing the efficiency in zinc immobilization.

As grains of the modifier are placed in a water environment with high concentrations of pollutants, different chemical and physical phenomena allow for immobilization. In the performed research, the mechanism behind immobilization efficiency focused on the adsorption capacity influenced by the specific surface area and grain porosity. Due to the high specific surface, materials with high adsorption potential (CHS, BC, AC, EAFIS 0/1, and NS) exhibited the ability to immobilize high quantities of zinc. In the case of organic modifiers, the grain structure allowed for the entrapment of pollutants within its vast pore network (Figure 8).

Except for the physical aspect of immobilization, the chemical phenomena associated with ion exchange significantly contribute to the efficiency of the entire process. The most effective inorganic modifier was EAFIS, an alkaline industrial by-product consisting mainly of CaO, MgO, FeO, and SiO_2_. It has been reported that its main mineral phases (alkaline oxides, calcium silicate, and calcium ferrite) can both form hydroxide precipitates between the OH- groups (hydrolysis of the alkaline substance) with various heavy metal ions and form stable heavy metal silicates and ferrites by ion exchange between Ca^2+^ (from calcium ferrite or silicate) and other heavy metal ions, thereby effectively removing heavy metal ions from the contaminated solution [56]. With precipitation from the solution, substances containing the aforementioned heavy metals are then adsorbed on the modifier’s surface due to its high specific surface (hence the difference in the immobilization efficiency between the same modifier of different granulations).

Not all considered modifiers exhibited immobilization potential. In the cases of coarse-graded EAFIS (1/2 and 2/4) and LFSAA, the exposure to a contaminated environment was limited due to their low specific surface areas. All of those were deemed to have insufficient properties to justify their use in cementitious composites in considered modification efforts and were not investigated further. The same could be said regarding copper slag. Its amorphous structure contributed to almost non-existent immobilization properties. Although the use of the modifier would not directly impact the properties of cementitious composites regarding non-mechanical properties of this nature, it was decided to include it in the second phase of the experiment, as copper slag can be accessed in significant amounts and is currently used to a limited extent in concrete technology. Also, it was considered that, although not having immobilization properties on its own, its addition could impact the characteristics of the cement matrix (its porosity and specific surface) and, therefore, the immobilization potential of the composite.

### 3.2. Properties of Modified Cementitious Composites

**Figure 9:**
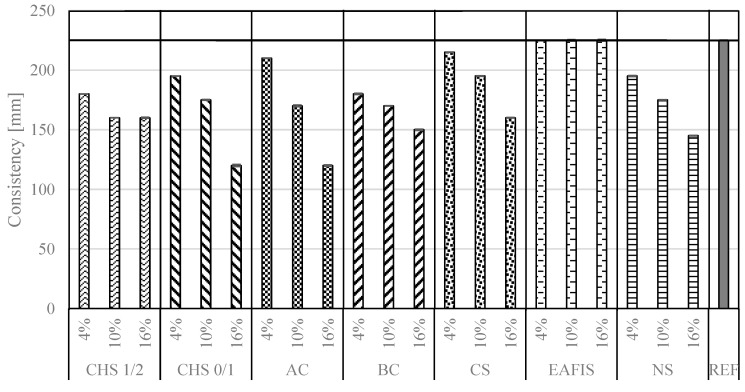
Consistency of fresh mortars modified with increasing mass amount of considered modifiers measured in a flow table test.

With changes in the composition of cementitious composite by replacing a part of the aggregate with a modifier, modifications to the rheology of the mix were observed. None of the investigated additions had a liquefying effect. The modification, depending on the origin of the modifier, either had no influence on it or contributed to an increase in the water demand of the mix (Figure 9). Most considered materials were characterized by either a high specific surface (nanoparticulate silica, fine-ground CSH, and EAFIS) or an extensive pore network (active carbon, and biochar) [57]. Those characteristics affect the distribution of mixing water throughout the composite, leading to a reduction in the availability of water required for maintaining consistency. As the experiment plan included a partial replacement of fine aggregate (0/2 Vistula river sand) with modifiers’ mass, and as its material characteristics regarding the potential for water adsorption and storage were significantly different from aggregate, an effect on rheological properties could be observed.

Porous filler materials are typically used to provide internal curing in cementitious composites, with the primary goal of reducing autogenous shrinkage by maintaining high internal relative humidity during binder hydration [3,10]. Various fillers, such as shells, wood pellets, and other organic materials, have been used for this purpose. However, when using additives with these properties, additional water must be added to the mix to compensate for the water initially absorbed by the modifier. Since this step was not included in the authors’ experiment, the porous grains in the mix reduced the available water, negatively affecting its workability. A significant reduction in water availability can lead to a severe deterioration in the mechanical properties of the hardened composite [10].

**Figure 10:**
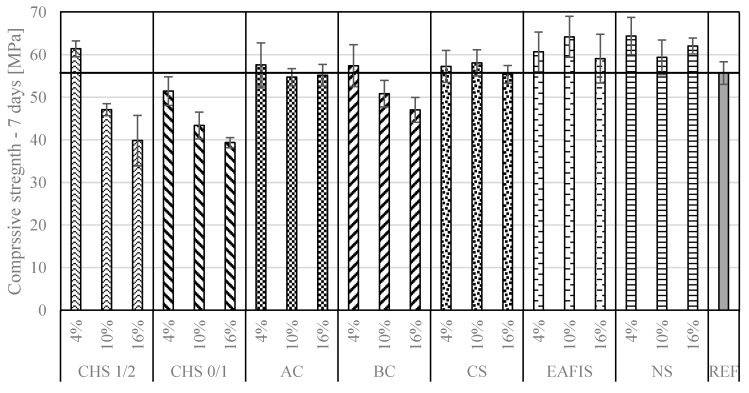
Compressive strength of hardened cement mortars modified with increasing mass amount of considered modifiers after seven days of curing.

**Figure 11:**
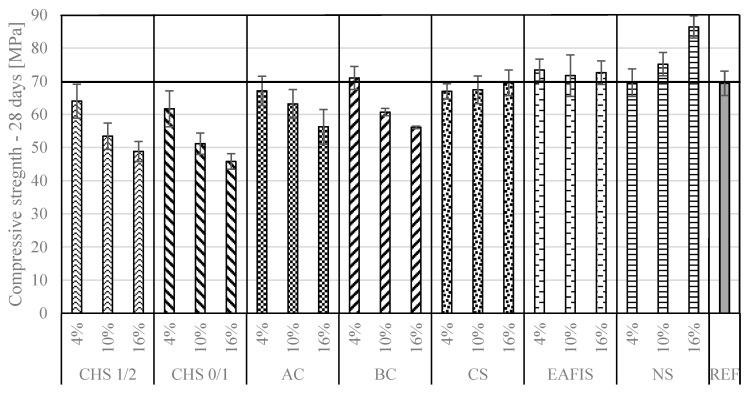
Compressive strength of hardened cement mortars modified with increasing mass amount of considered modifiers after 28 days of curing.

The mechanical performance varied among the considered mortars. Adding organic fillers (CHS, biochar, and active carbon) contributed to significant deterioration in compressive strength alongside an increase of modifiers mass in the composition (Figure 10 and Figure 11). The effect was most intense in the case of replacing 16% of fine aggregate’s mass with CHS 0/1—the compressive strength decreased by 36% from approx. 70 MPa to approx. 45 MPa after 28 days of curing.

A distinction between the modifier’s origin and its influence over compressive strength could be observed—all organic modifiers contributed to a deterioration in the mechanical performance of the composite, while all considered inorganic modifications allowed for the maintenance or slight increase of the compressive strength, especially after 28 days of curing. That was the case for nanoparticulate silica modification, where, by replacing 16% of the fine aggregate’s mass, the compressive strength of the composite increased by approx. 21% from approx. 70 MPa to approx. 85 MPa after 28 days of curing. The modification with copper slag and EAFIS did not significantly impact the compressive strength after 28 days (ANOVA, *p* >> 0.05). A similar observation was made for compressive strength after 7 days—for mortars modified by AC, CS, EAFIS, and NS, an increase in the content of each of these modifiers within the tested range did not significantly affect the compressive strength of the mortars after 7 days (ANOVA, *p* >> 0.05).

The kinetics of binder hydration also varied between modifiers. An increase in compressive strength between 7 and 28 days of curing was the lowest in the case of organic modifications, suggesting a significant disturbance in the hydration process due to the limited availability of mixing water adsorbed by modifiers’ grains and significant changes to pore network characteristics of the composite. In the cases of EAFIS and CS, the hydration kinetics were within the same range as for the reference series. It indicated a non-significant influence of those modifiers on the binder hydration. As all of the considered modifications were not characterized by binding properties, their influence over hydration was considered to be over water transport (adsorption and absorption) during hardening, contributing to changes in water availability during hydration.

**Figure 12:**
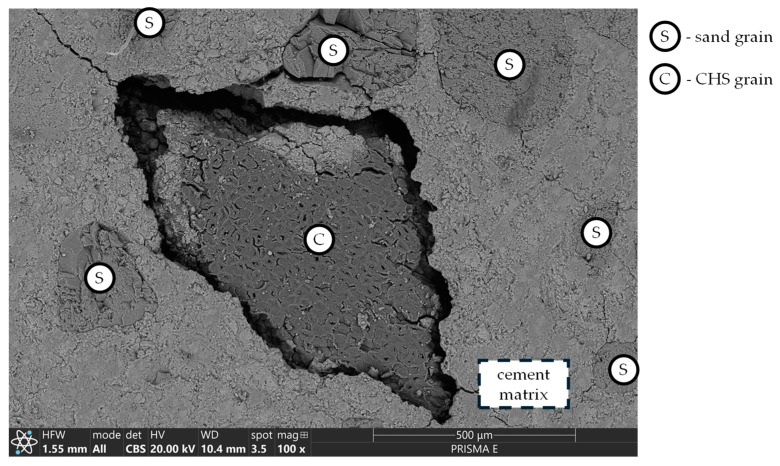
The lack of interim layer (SEM BSE) between CHS grain and cement matrix. No apparent connection can be observed over the two phases of the composite.

The compatibility with cement matrix varied between organic fillers—CHS, a material initially intended for isolating its interior from the external environment, was characterized by low water absorption (in the case of the CHS used in this research, its maximal water absorption capacity was determined to be 0.654 g/g after 24 h of exposure to a water environment). Additionally, since CHS has different thermal expansion properties compared to the cement matrix, temperature changes and fluctuations in internal relative humidity during cement hydration, as well as during sample preparation for subsequent tests, caused differential expansion or contraction between the cement matrix and the shells. This mismatch led to internal stresses within the composite, ultimately resulting in cracks. It contributed to a fragile interlayer between CHS grains and the cement matrix (Figure 12), which did not provide sufficient compatibility between two layers of different origins. This effect contributed to various changes over different composite properties, significantly affecting the connectivity of the entire composite pore network.

**Figure 13:**
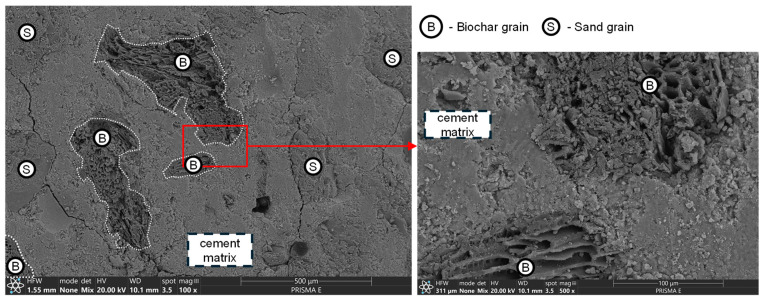
No visible distinct boundary (SEM BSE/SE mix mode 50–50%) between the biochar grains and the cement matrix, along with magnification of the indicated area.

This was not the case when investigating biochar and active carbon modifications. A crystallized interim layer between the modifier’s grain and cement matrix was observed (Figure 13). Unlike CHS, carbon-based modifiers had extensive grain pore networks and high water absorption potential (in the case of the biochar used in this research, its maximal water absorption capacity was determined to be 2.479 g/g after 24 h of exposure to a water environment, for active carbon—1.107 g/g). During mixing with other ingredients of mortar, a portion of mixing water was adsorbed on the surface of the modifier’s grains, allowing for the undisturbed hydration of the binder in its proximity and a sufficient coupling of the grain with cement matrix (high specific surface area on which the crystallization of hydration products occurred). The mechanical properties of the modifier itself caused the deterioration in the mechanical performance of the mortar. Due to the material’s brittleness, higher biochar dosages increase the likelihood of microcracks within the cement matrix, weakening the overall strength of the composite [58].

**Figure 14:**
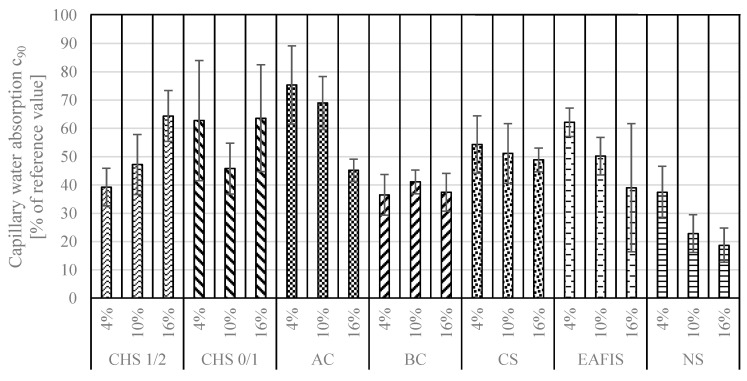
Capillary water absorption coefficient c90 of modified mortar series represented as a percentage of a reference value.

**Figure 15:**
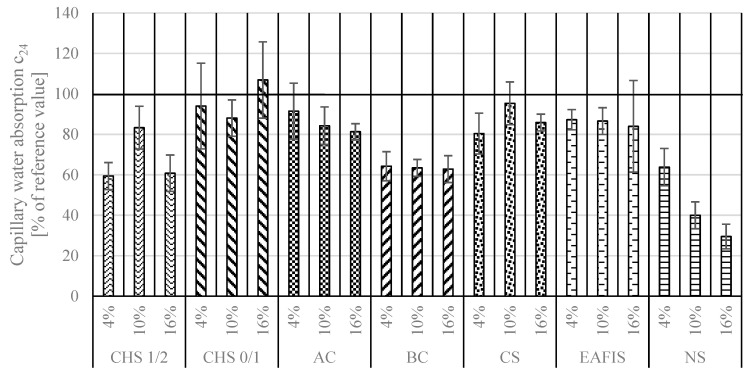
Capillary water absorption coefficient c24 of modified mortar series represented as a percentage of a reference value.

According to the aforementioned standard, capillary water absorption was investigated after 10 min, 90 min, and 24 h. Based on the performed research, two values describing the connectivity of the pore network were calculated—capillary absorption c_90_ [kg/m^2^min^0.5^] (the change in water mass absorbed by mortar sample between 10 and 90 min of immersion in distilled water) and c_24_ [kg/m^2^] (based on the difference in the mass of the dried sample before the test and after 24 h of immersion in distilled water). Considered modifications caused significant reductions for both coefficients—all contributed to changes in the continuity of the pore network (Figure 14 and Figure 15). This effect was primarily caused by the experiment design—a part of the fine aggregate was replaced with various amounts of different modifiers, usually of a finer granulation than that of Vistula sand. As a result, a sealing effect of the pore network could be observed. The nanoparticulate silica modification contributed to the most significant reduction in capillary water absorption—a series in which 16% of the fine aggregate mass was replaced with nanoparticulate silica was characterized by a c_90_ coefficient approx. five times lower than that of the reference series (a reduction from 0.1825 to 0.0342 [kg/m^2^min^0.5^]), with a c_24_ coefficient of approx. four times lower (a decrease from 2.5781 to 0.7604 [kg/m^2^]).

However, increasing the content of modifiers within the adopted range did not always lead to a decrease in the capillary suction of modified mortars. For example, increasing the content of CHS, BC, and CS from 4% to 16% did not significantly affect capillary water absorption in the modified mortars after 90 min (ANOVA, *p* >> 0.05). On the other hand, the capillary water absorption of mortars containing these modifiers differed significantly after longer exposure—24 h. Only the results obtained after 24 h from the samples modified with EAFIS and BC did not show significant variation (ANOVA, *p* > 0.05).

Except for reducing the porosity of the composite due to a different granulation, the grains of the considered modifications introduced an additional variable influencing the water transport through the pore network of the composite. Similar to the mechanism of internal curing of the cementitious composites, depending on the properties of modifier grains regarding water absorption potential and specific surface area, hydrostatic pressure distribution within the pore network can vary and significantly impact its water transport properties [59]. Although CHS, BC, and AC contributed to significant deterioration in the mechanical performance of the composite (suggesting an increase in the pore volume of the cement matrix), the capillary water absorption remained lower than in the case of the reference series. The trend was most significant in the case of modification with active carbon—with an increase in mass content of AC in the composite, a decrease in its water absorption characteristics was observed, reaching less than half of the c_90_ coefficient and 80% of c_24_ of the reference series. Regardless of the BC content in the mortar, it also contributed to a significant reduction in capillary water absorption.

The sealing effect was not observed in the case of CHS 0/1 modification. The authors believe that the negative impact of the addition of the aforementioned modifier on the mechanical performance of mortar was caused by a significant increase in the porosity of the cement matrix. In the case of CHS 1/2, those changes were locally distributed and limited to the volume of the composite in the direct contact of CHS grains. As the granulation of CHS was reduced to 0/1, the number of fine-graded grains significantly increased, enhancing the connectivity of the pore network (visualized in Figure 12).

### 3.3. Zinc Immobilization Efficiency and Pore Network Characteristics

**Figure 16:**
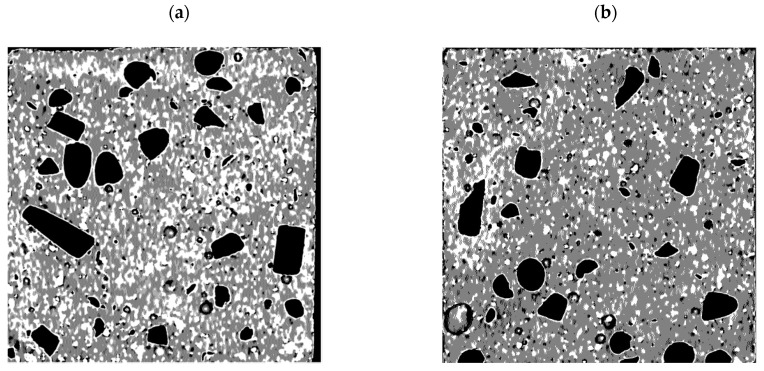
Macroscans (monochrome) of samples prepared for immobilization tests with exposed active carbon (AC) grains (black). Irregular distribution of grains (amount, surface) was observed between different samples from the same series for all coarse-graded modifications; (**a**,**b**)—external surface of different specimens cut from the same mortar sample.

The immobilization properties of the investigated mortars were influenced by the area of modifier grains exposed to the external environment during sample preparation. For coarse-graded modifications, this exposed area varied significantly between samples within the same series (Figure 16), leading to high variability in the immobilization test results. This was due to the differing orientations of coarse grains in various cross-sections of the samples, as well as the relatively small sample size used for the tests (40 mm × 40 mm × 10 mm) compared to the diameters of the coarse-graded modifier grains. This phenomenon was not observed in the fine-graded modifications, except for CHS 1/2. In that case, due to thermal incompatibility with the cement matrix (Figure 12), the sample preparation process (involving mechanical cutting, washing, and drying at 105 °C) dislodged some exposed CHS grains, with the amount varying between samples. Since the immobilization mechanism depended on the accessibility of pollutants to the surface of the modifier grains as well as the cement matrix, changes in the exposed area across different samples led to variations in their immobilization effectiveness.

**Figure 17:**
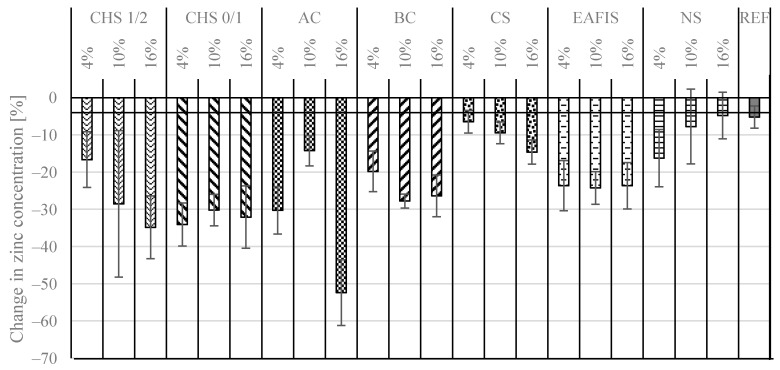
The zinc immobilization potential of modified and reference mortars was determined through the FAAS tests of zinc concentration in a water solution before and after its exposure to cementitious samples for 15 min.

It was found that depending on the type of applied modification, the mortar’s ability to immobilize zinc varied (Figure 17). The lowest potential in zinc removal was recorded for the reference series—the concentration of Zn reduced slightly by 5.2%, from 84.6 mg/L to 80.2 mg/L after 15 min of exposure. The most effective in this regard were organic modifications (CHS, biochar, and active carbon). However, the effectiveness of all aforementioned modifications was characterized by high standard deviations. The most promising were the modifications with fine-graded materials, as the granulation allowed for their homogenous distribution within the entire cement matrix, allowing for an increase in repeatability in the results conducted for different samples from the same series. It was found that the effectiveness of immobilization did not increase linearly with an increase in the mass content of the modifier (CHS 0/1 and EAFIS 0/1). It suggested an additional influence of modifier grains on the immobilization properties.

An increase in the content of considered modifiers usually significantly affected the mortar’s ability to reduce the concentration of zinc ions in the examined solutions (ANOVA, *p* < 0.05). However, for CHS 0/1, BC, and EAFIS, the zinc immobilization efficiency remained the same regardless of the amount of additive in the mortar.

With the introduction of an additional organic or inorganic phase into the cement matrix, different phenomena contribute to an increase in the immobilization properties of the composite. Apart from the physical and chemical characteristics of the grain’s surface, which can facilitate processes influencing heavy metal removal rate, a biological aspect should also be considered. Iron oxides of different origins and most organic fillers expedite the appearance of biofilms on the surface of the composite exposed to the external environment. With their presence, bio-immobilization and/or bio-reduction further increase the immobilization properties of the composite [60]. Although the performed research did not focus on the bio-mechanisms of heavy metal removal, and the developed method did not expedite the appearance of bio-films on the cement matrix, it has been reported that this aspect significantly influences the properties of pervious concrete regarding immobilization properties over time [61,62].

**Figure 18:**
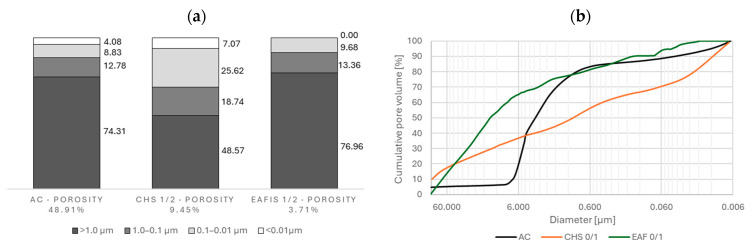
(**a**) Total porosity and approximate size distribution of pores of selected modifiers—active carbon (AC), crushed hazelnut shells (CHS), and electric arc furnace iron slag (EAFIS); (**b**) size distribution of pores of selected modifiers—AC, CHS, and EAFIS.

As an additional filler is introduced into the composite, due to its different characteristics regarding porosity and water absorption, the density and overall porosity of the cement matrix within the direct proximity of its grains would be impacted. Most immobilization phenomena focus on the cement matrix’s chemical and physical impact on the characteristics of an environment from which pollutants are immobilized on its surface. The parameters of it are affected by the modifier presence and, in effect, the specific surface on which those phenomena occur. Combined with an extensive porosity of modifier grains, it can significantly increase immobilization potential (Figure 18).

The most porous modifier for materials with high immobilization potential was active carbon (AC), in which 48.91% of the grain volume constituted a pore network. Although crushed hazelnut shells (CHS) were characterized by much lower total porosity (9.45%), the distribution of pores of different diameters varied significantly from other organic modifiers—more than 30% of total pore volume constituted pores of diameters below 0.1 µm. Electric arc furnace iron slag (EAFIS) was characterized by low total porosity, not exceeding 4%. Also, more than 75% of its pore volume was constituted by pores of diameters exceeding 1.0 µm (macropores). The modifiers’ porosity characteristics were accessed using mercury intrusion porosimetry (MIP) on modifiers’ grains exceeding 1 mm in diameter. It was assumed that CHS and EAFIS of finer granulation had approximately the same pore characteristics as their coarser variants. Fine-graded modifiers (of 0/1 granulation) were not investigated via MIP, as the authors wanted to avoid the risk of contaminating the apparatus with fine grains while applying negative pressure to the penetrometer filled with tested material prior to it being filled with mercury.

**Figure 19:**
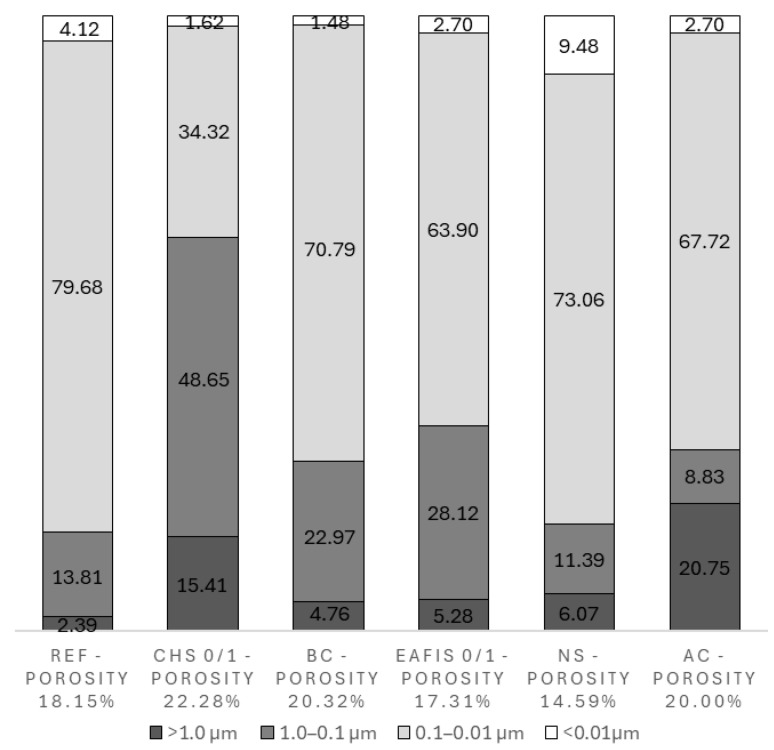
Total porosity and volumetric content of pores of various diameters in the total pore volume of cement mortars with 16% of fine aggregate mass being replaced with selected modifiers.

The proposed modifications significantly impacted the pore network characteristics of the cement matrix. In all investigated cases, except in nano-silica and EAFIS 0/1 modification, the total porosity of mortars increased compared to the reference variant, to an extent dependent on the porosity of the modifier. The highest total pore content was observed for CHS 0/1 modification, 22.28%, compared to 18.15% for the reference series (Figure 19).

The distribution of pores of different diameters also varied. All organic modifications contributed to an increase in the number of pores of diameters exceeding 0.1 µm. This case was most notable for CHS modification, as more than 50% of pore volume constituted pores of the aforementioned diameters. Biochar and active carbon had a comparable effect on the pore network characteristics and total pore volume. However, AC modification contributed to a significant increase in macropore content (above 1.0 µm), exceeding its value of reference series approximately tenfold. EAFIS and nano-silica modification contributed to a reduction in the total pore volume of the cement matrix. An effect of the pozzolanic reaction between nano-silica and calcium hydroxide resulted in the cement matrix’s densification, causing the matrix’s overall porosity to reduce significantly (from 18.15% to 14.59%).

**Figure 20:**
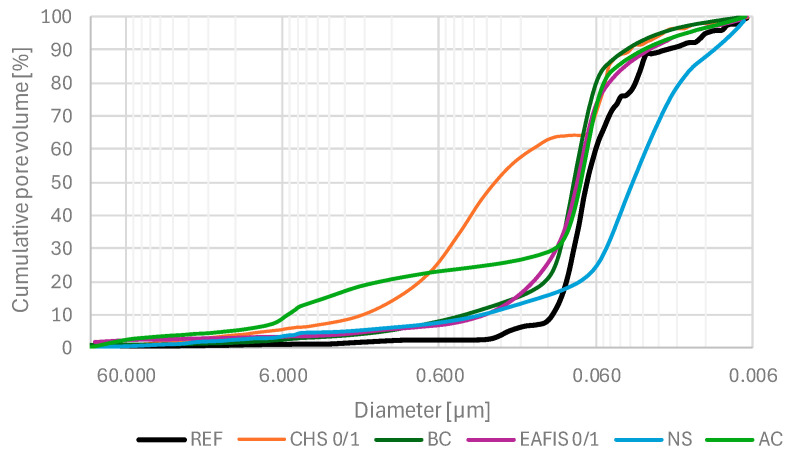
Cumulative pore volume diameter curves of selected cement mortars investigated in the study through the mercury intrusion porosimetry technique.

This effect was also noticeable in the case of the relative volume of pores of diameters below 0.1 µm, as NS modification was the only one that contributed to a significant increase in its content compared to the reference series (Figure 20). Most significant changes regarding the content of macropores were observed in the case of CHS 0/1 and AC modifications. In the case of AC, pores of diameters above 1.0 µm constituted more than 20% of the total pore volume. The effect of an increase in the porosity of the matrix was even more significant in the case of CHS 0/1 modification, as, except for similar content of pores of diameters above 1.0 µm, the volume of pores with diameters above 0.1 µm exceeded 60% of total pore volume (a more than tenfold increase compared to the reference series).

As previously shown, all modifications that contributed to the deterioration of the composite’s mechanical performance (crushed hazelnut shells, activated carbon, and biochar) caused significant changes to the porosity of the cement matrix, increasing its macropore content. For example, the modification with CHS 0/1 16% increased the proportion of pores larger than 1 µm in diameter from 2.39% to 15.41%. Additionally, these modifications increased the total porosity of the cement matrix, with the CHS 0/1 16% modification raising the total porosity from 18.15% to 22.28%. An increase in the pore network volume within the composite typically leads to a reduction in mechanical performance, as it reduces the volume through which stress can be transferred within the composite’s structure. This was observed in the research, further exacerbated by the lack of proper compatibility between certain modifications (such as CHS) and the cement matrix.

**Figure 21:**
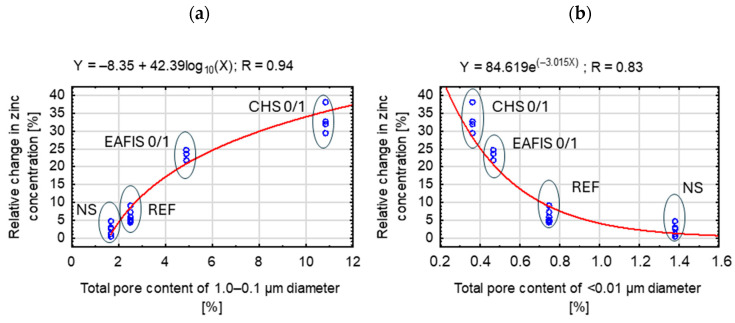
Statistically significant dependence between the total content of pores of specific diameters and the zinc immobilization potential of non-carbon-modified mortars; (**a**) dependence between capillary pore content and zinc immobilization potential; (**b**) dependence between gel pore content and zinc immobilization potential.

It was found that for conducted modifications, except carbon-based modifiers, a trend could be found, linking the volumetric content of pores of specific diameters and the zinc immobilization effectiveness of the composite (Figure 21): 1.0–0.1 µm (capillary pores) and <0.01 µm (gel pores). The properties of the cement matrix are one of the factors influencing the immobilization potential of the composite. The rest of those focus on the properties of the immobilization modifier.

In the cases of both AC and BC, the positive impact of the modifier’s properties over the immobilization efficiency tested in the study surpassed that of the cement matrix, making its influence over the overall performance of the composite less statistically relevant—the exceptional immobilization properties of the aforementioned modifiers caused an increase in the immobilization effectiveness, surpassing that arising from the modifier’s influence over the pore network.

However, in the case of other modifiers, a trend could be observed, linking the total volume of pores of specific diameters with the immobilization effectiveness of the cement matrix. In the case of gel pores (<0.01 µm), an increase in their total volume indicates an overall decrease in the total porosity of the cement matrix. That was the case for NS modification, which reduced the adsorption area due to the NS sealing effect (increase in the gel pores content at the expense of pores of larger diameters) over the pore network of the composite. Due to the pozzolanic activity of the modifier, it reduced the effectiveness of zinc immobilization while increasing the mechanical performance (reduction in the total porosity of the composite due to the crystallization of reaction products between NS and hydroxides in the cement matrix). A different trend was observed for EAFIS and CHS modifications. In the case of CHS, the composite’s overall porosity and the content capillary pores increased, suggesting a significant influence of the modification on the course of the hydration of the binder, verified through the mechanical tests of prepared composites. All aforementioned changes to pore distribution and total volume contributed to a significant increase in zinc immobilization effectiveness. Iron slag addition reduced the overall porosity of the composite. However, the distribution of the remaining pores changed significantly, increasing the content of capillary pores within the cement matrix and, as a result, the range and scope of capillary suction.

**Figure 22:**
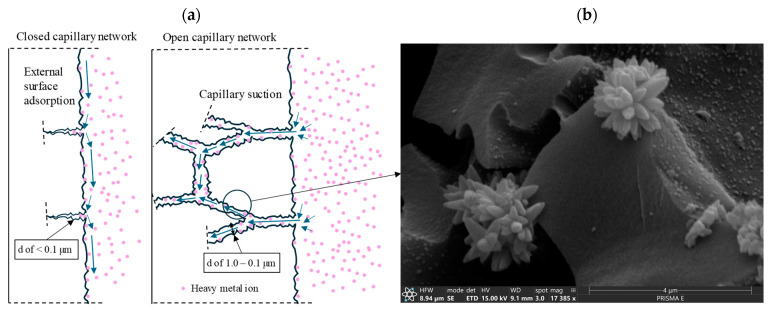
(**a**) Schematic for the influence of pore network characteristics on the efficiency of heavy metal immobilization and (**b**) SEM micrograph of a crystallized grain within modifier’s capillary pore network.

Depending on their size and connectivity, different types of pores can either facilitate or hinder the transport of fluids within the cement matrix. Capillary pores, which are larger and more interconnected, enhance immobilization by providing space for diffusion, physical entrapment, and precipitation. These larger pores allow easier access to hydration products and promote long-term immobilization as the matrix densifies over time. The relatively open structure of capillary pores facilitates the transport of zinc ions and enhances their interaction with the matrix, making them more effective at capturing zinc (Figure 22).

In contrast, gel pores are much smaller, and their limited size and lower connectivity restrict ion diffusion, reducing access to the internal matrix. Although gel pores offer a high surface area for potential adsorption, their small volume hinders precipitation and immobilization. As a result, a higher content of gel pores in the cement matrix reduces the overall efficiency of zinc immobilization, while a greater proportion of capillary pores improves fluid accessibility to the surface of the cement matrix, where various ions can be immobilized.

However, while a well-developed capillary pore network is beneficial for pollutant immobilization, excessive capillary porosity in the cement matrix negatively impacts the mechanical performance and durability of the composite. This was particularly evident in the mechanical performance of the cementitious composites modified with organic materials, which increased both overall porosity and capillary pore content. The introduction of a continuous pore network, especially when exposed to external environmental conditions, leads to a loss of durability in cementitious composites due to phenomena such as freeze–thaw cycles and the ingress of corrosive agents driven by water transport.

It is expected that all considered organic modifications would contribute to significant deterioration in the mechanical performance of the composite over time. The iron slag modification slightly increased the capillary pore content relative to the reference composite. However, the modified mortar was characterized by similar mechanical performance, and steel slag grains were compatible with other elements of the cement matrix, reducing the number of interconnected defects in the cement matrix.

**Figure 23:**
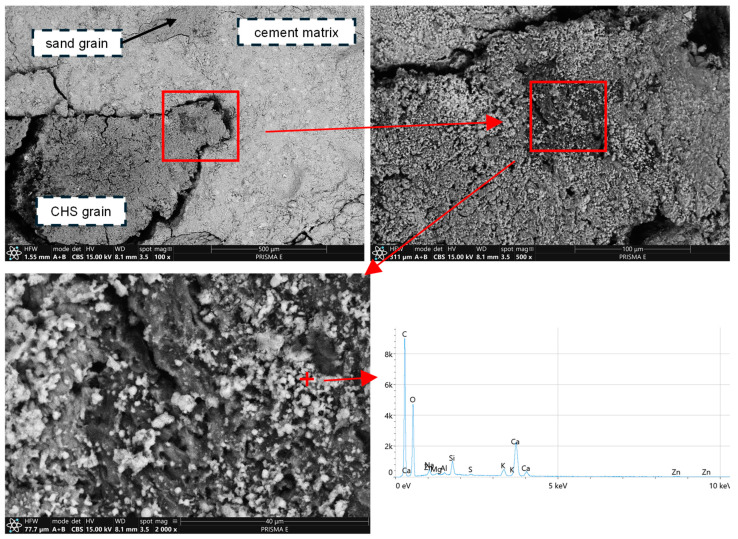
Zinc physical adsorption (SEM BSE) on the exposed surface of CHS grain, along with EDS results from selected point indicating the presence of zinc-related chemical compounds.

With its significant impact on the cement matrix’s properties and high internal porosity, CHS 0/1 modification allowed for zinc immobilization efficiency to increase significantly compared to the reference series. CHS grains constituted an ideal environment for zinc immobilization due to an extended pore network of specific diameters (Figure 23).

The main phenomena contributing to the stabilization/solidification mechanism for heavy metal immobilization include adsorption on the surface, chemical bonding, and physical encapsulation [63,64]. Through absorption, heavy metal ions adhere to a specific surface (no chemical reactions). Afterward, the chemical bonding phase includes various chemical reactions between heavy metal ions and other constituents of the surface environment (for example, precipitation in the alkaline environment, ion exchange with hydration products, or surface complexation) [63]. The final phenomenon is physical encapsulation, which includes heavy metal precipitation or complexes to fill pores of specific volume, which are later encased with products of different hydration reactions. In alkaline environments, Zn^2+^ ions precipitate or complex mostly as Zn(OH)_2_, ZnCO_3_, ZnO, Zn_3_(PO_4_)_2_·4H_2_O, and CaZn_2_(PO_4_)_2_·2H_2_O [65]. If the modifier is characterized by specific pore network characteristics, soluble hydration products are going to crystallize within its structure, which afterward provides an effective immobilization environment for heavy metal contaminants. That was the case for all organic modifications, and the same phenomena were observed for AC and BC—modifier grains accelerated the phenomena linked with zinc immobilization.

### 3.4. Thermogravimetric Analysis

**Figure 24:**
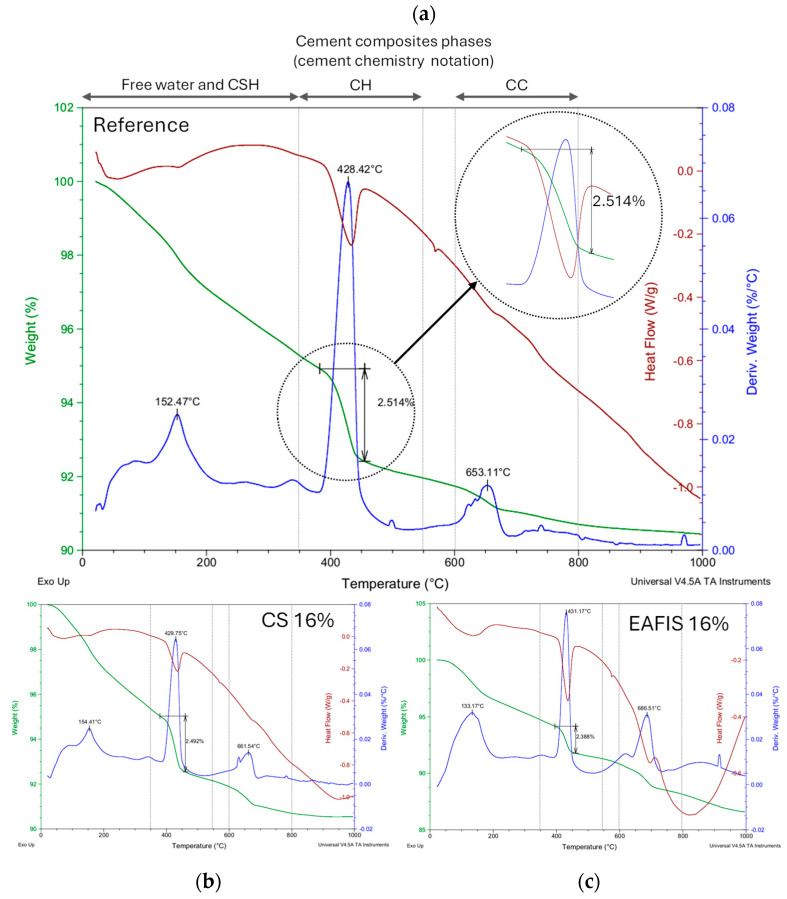
Thermogravimetric analysis for (**a**) reference mortar and versions of it modified with inorganic modifiers (**b**) CS—copper slag, (**c**) EAFIS—electric arc furnace iron slag 0/1. The magnified area indicated the fragment of TGA’s curve from which the portlandite content was calculated.

TGA–DSC analysis was performed to establish the influence of the selected modifiers on the phase composition of the modified mortars. It was found that the course of thermal decomposition did not vary significantly between non-modified mortar (reference) and mortars modified with copper slag (CS) or electric arc furnace iron slag (EAFIS) (Figure 24), regardless of the amount of modifier. A significant mass loss was observed for three distinct temperature ranges: below 350 °C (associated with C-S-H thermal degradation), 350–550 °C (related to thermal decomposition of CH—calcium hydroxide), and 600–800 °C (associated with the thermal decomposition of C—carbonate minerals; mainly calcite and dolomite) [66].

By knowing that the molar mass of water (approx. 18 g/mol) and the mass loss of the sample during TGA analysis, caused by evaporation of water from calcium hydroxide (CH), the amount of calcium hydroxide (approx. 74 g/mol) content in the samples could be calculated (Equations (1) and (2)) [66], where M_1_—mass of the sample at the temperature, which corresponds to the beginning of CH decomposition; M_2_—mass of the sample at the temperature which corresponds to the end of CH decomposition; and M_0_—initial mass of the sample.

(1) $$\underset{74\;g}{\underbrace{{Ca(OH)}_{2}}}\to \underset{56\;g}{\underbrace{CaO}}+\underset{18\;g}{\underbrace{H_{2}O}}$$

(2) $${Ca(OH)}_{2}=\left(\frac{M_{1}-M_{2}}{M_{0}}\right)\times 100\%$$

It was confirmed that the aforementioned additions (EAFIS and CS) did not participate in the hydration of the binder and exhibited chemically inert filler functions (Table 2), as opposed to nano-silica (NS) and crushed hazelnut shells (CHS). Samples modified with NS and CHS were characterized with a significantly lower amount of portlandite than the reference sample. In the case of nano-silica, it could be explained by the pozzolanic reactions between reactive silica and portlandite, described extensively in the literature [67,68,69,70].

Hazelnut shells mainly consist of lignin (40–51%), hemicellulose (13–32%), and cellulose (17–27%) [71,72,73,74,75]. Calcium ions form compounds with lignin, as Ca(OH)_2_ blocks its easily ionizable functional groups, such as the carboxyl group, the methoxy group, and the hydroxyl group [76,77]. Those reactions contribute to a reduction in the overall content of portlandite in the cement matrix, in some cases, reducing its content to an amount comparable with NS modification.

The solubility of heavy metal ions is negatively correlated with an increase in pH [31]. However, in the conducted study, its effect on zinc immobilization was overcome by other characteristics of the investigated composites—mainly the porosity of both modifiers and the pore network characteristics of cement matrix that was exposed to a zinc-rich environment. The non-significant influence of the aforementioned parameter was most evident in the case of the reference cementitious composite and series modified with copper slag. Both mortars were characterized by the highest content of Ca(OH)_2_. However, their immobilization potential was one of the lowest of all the considered modifications. It suggests that the surface properties of the cement matrix had significantly more impact on the composite’s potential to immobilize pollutants.

**Figure 25:**
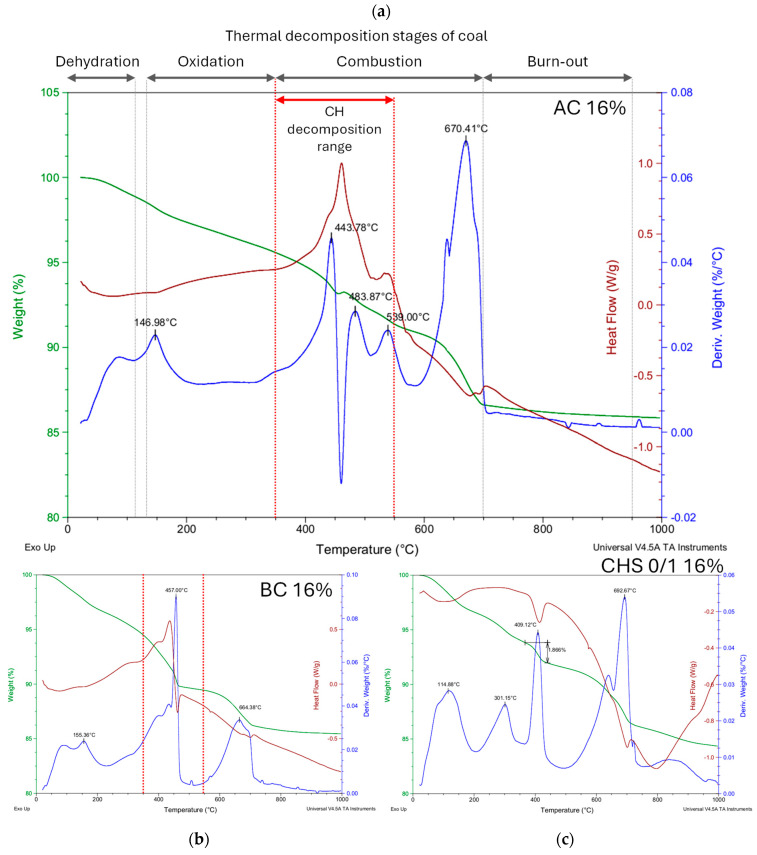
Thermogravimetric analysis for mortars modified with organic modifiers (**a**) AC—active carbon, (**b**) BC—biochar, and (**c**) CHS—crushed hazelnut shells 0/1.

Analysis regarding the Ca(OH)_2_ content could not be viably performed for other organic modifiers. For the series modified with biochar (BC) and active carbon (AC), due to the origin of the modifiers, other effects linked with thermal decomposition interfered with the proposed calculation model. Unlike for cementitious composites, the thermal decomposition of coals can be divided into four stages, which overlap with those typically occurring in standard cement systems: coal dehydration/desorption (30 to 110 °C), oxidation (130 to 350 °C), combustion (350 to 700 °C), and burnout (700 to 950 °C) [78,79]. Due to the fact that the mass loss of the carbon-modified sample is caused by the decomposition of compounds of different origins in the same temperature range as Ca(OH)_2_, it would be highly inaccurate to approximate the portlandite content based solely on TGA analysis. However, the influence of the aforementioned modifiers on the course of the thermal decomposition of the modified mortars is distinguishable from the reference cement mortar (Figure 25).

## 4. Conclusions

Cementitious composites can be used for purposes other than purely mechanical, positively affecting human comfort and environmental aspects. The cement matrix’s heavy metal immobilization characteristics can be enhanced by incorporating various modifications, including by-products of other industrial processes. Based on the conducted research, several of the following conclusions regarding water treatment through its exposure to cementitious composites could be reached.

- Pore network characteristics of the cement matrix have a significant influence over its immobilization properties, especially in the case of non-carbon modifiers—modifications contributing to an increase in the content of capillary pores through which the transport of fluids can occur were characterized by significantly higher immobilization potential;
- An increase in the capillary pore content, although proven significant in the case of the pollutant immobilization, contributed to significant deterioration of the mechanical performance of cementitious composites—it is highly probable that it also contributed to a decrease in the durability of the investigated composites (ease of access of corrosive agents into the cement matrix);
- The modification through electric arc furnace iron slag (EAFIS) has significantly improved immobilization characteristics of the cement matrix without determining the mechanical performance of the composite—its impact on the rheological properties was also marginal;
- Except for the modifier’s origin and characteristics, its granulation (specific surface) has a significant influence over immobilization characteristics—fillers with coarser granulation of the same origin exhibited reduced immobilization efficiency of heavy metals;
- Cementitious composites modified by organic fillers (biochar, active carbon, and crushed hazelnut shells) contributed to the highest efficiency in immobilization. However, due to the highest increase in the porosity of the cement matrix, their effect over other characteristics of the investigated mortars was most detrimental from all considered modifications;
- The addition of organic fillers (active carbon, biochar, and crushed hazelnut shells) contributed to a significant deterioration of the rheological properties of the investigated composites—this effect could be mitigated by adopting a similar approach as in the case of internal curing agents for cementitious composites (increase in the total water content in the mix) or through the use of rheology-modifying admixtures;
- Organic modification with crushed hazelnut shells resulted in a lack of proper compatibility between cement matrix and filler grains. Although it contributed to an increase in the immobilization potential, the durability of the composite significantly deteriorated;
- Modification with nanoparticulate silica did not contribute to an increase in immobilization properties—its effect over the pore network resulted in a significant decrease in the porosity of the cement matrix, significantly reducing the capillary suction within the pore network, severely reducing its immobilizing specific surface area and, therefore, its adsorption surface.

The capacity for the immobilization of cementitious composites exposed to heavy metal environments is finite. Although concentrations of various pollutants in urban surface runoff are relatively low, it is expected that at some point in the service life of a cementitious composite, it either would require maintenance efforts to restore its immobilization potential or would need to be disposed of. The authors intend to investigate if, and to what extent, it is possible to manipulate the immobilization capacity of various pollutants and how to maintain those properties over time, as well as how the increased content in various immobilized pollutants affects the recycling potential of such composites.

## Figures and Tables

**Table 1:** Properties of the binder used in the study.

Characteristics	Unit	Value
Tensile strength, two days	MPa	4.74
Tensile strength, 28 days	MPa	4.46
Compressive strength, two days	MPa	23.84
Compressive strength, 28 days	MPa	44.41
Initial setting time	min	190
Final setting time	min	270
Specific gravity	g/cm^3^	3.09
Specific surface area	cm^2^/g	3920

**Table 2:** Calculated calcium hydroxide content based on TGA analysis (significant reductions marked with gray color).

Type of Modifier	Modifier Content [% m.a. ^1^]	Mass Loss [% m.s. ^2^]	Ca(OH)_2_ Content [% m.s. ^2^]
Reference	0	2.514	10.27
Copper slag (CS)	4	2.610	10.74
	16	2.492	10.26
Nano-silica (NS)	4	1.993	8.20
	16	1.529	6.39
Crushed hazelnut shells 1/2 (CHS 1/2)	4	1.960	8.05
	16	1.829	7.53
Electric arc furnace iron slag 0/1 (EAFIS)	16	2.388	9.80
Crushed hazelnut shells 0/1 (CHS 0/1)	16	1.866	7.68

^1^—mass of aggregate. ^2^—mass of sample.

## Data Availability

Data available on request.
